# Pneumothorax with liposuction, awareness of rare complication: a case report

**DOI:** 10.1186/s13256-023-04250-z

**Published:** 2023-12-09

**Authors:** Abdulelah Alwadai, Mustafa Gaber, Alwaleed Khalid Alammar, Abdullah M. Alsaedan, Jamal Eldaib

**Affiliations:** 1 Department of Plastic Surgery and Burn Unit, Aseer Central Hospital, Abha, Saudi Arabia; 2 Department of Plastic Surgery and Burn Unit, Prince Sultan Military Medical City, 11159 Riyadh, Saudi Arabia

**Keywords:** Case report, Complication, Liposuction, Pneumothorax

## Abstract

**Background:**

Liposuction is the most commonly performed procedure in aesthetic plastic surgery worldwide, the complications and morbidity are under evaluated. Pneumothorax is thought to be a rare complication after liposuction but the exact rate still unknown.

**Case presentation:**

We presented to you a 45-year-old Arabian female with history of hypertension underwent lipoabdominoplasty, back liposuction and gluteal lipofilling. On the first postoperative day, the patient was complaining of chest pain accompanied with tachypnea and tachycardia, oxygen saturation was maintained on room air. Upon chest auscultation, diminished air entry was markedly noted on her left side, immediate chest x-ray and electrocardiogram (ECG) was done, which showed unremarkable x-ray and ECG shows sinus tachycardia. Computed tomography (CT) carried out and showed left side pneumothorax. An urgent thoracic surgery consultation was done and chest tube was inserted. The patient reported immediate improvement of her symptoms and the vital signs retain to normal range. On day 3, air leak stopped, chest tube was clipped by thoracic surgery, and the chest tube was removed 24 h later. The patient had a relatively smooth recovery with no other complications.

**Conclusion:**

Pneumothorax have possibility to happen with liposuction, awareness of possible risk factors should detect by plastic surgeon, to manage earlier as soon as possible.

## Introduction

Pneumothorax is a common medical condition, its defined as presence of gas within the pleural space [1]. In the setting of plastic surgery, it considered a rare complication that can be occurred due to several surgical procedures, including breast augmentation and upper trunk liposuction [2]. Pneumothorax may occur spontaneously or secondary to multiple causes such as, infection, malignancy, or direct trauma to the lung or chest wall [1]. In a surgical setting, iatrogenic pneumothorax is caused by intraoperative tear of the pleura, needle puncture with injection of local anesthetic, or as result of forced air entry into the pleural space due to high pressure in the surgical pocket causing barotrauma [2].

## Case presentation

A 45-year-old Arabian female with history of controlled hypertension on Co-Diovan 160\12.5 mg (Valsartan\hydrochlorothiazide), presented with a complain of abdominal laxity, the physical exam showed abdominal laxity, diversion of recti, upper back lipodystrophy. Patient have been counseled about lipoabdominoplasty, back liposuction and gluteal lipofilling. The procedure was done under general anesthesia; vital signs and all parameters were within the normal range throughout the procedure and early recovery period. Power-assisted liposuction (PAL) using MicroAire (Charlottesville, Va.) and ultrasound assisted liposuction (UAL) using VASERlipo were used in this case. Infiltration was done using cannulas that are size 4 in diameter (Evamatic, by Euromi) with super wet technique. The procedure started with supine position for lipoabdominoplasty with liposuction of flank and waist. Longitudinal plication and oblique transverse plication to defined the waist in better shape. Then, the patient was turned to prone position for the completion of back, lateral gluteal depression liposuction as well as the gluteal area. In gluteal lipofilling, local infiltration was done using syringes along the incision sites, and cannulas size 4 were used. Pressure garment was applied post operative, and nothing unusual occurred throughout the procedure which took nearly 6 h. VTE (venous thromboembolism) prophylaxis included: adequate hydration, early ambulation, elastic stockings, and chemoprophylaxis with Low-molecular-weight heparins (LMWHs) (Enoxaparin 40 IU, subcutaneous, 8 h after the procedure and for 4 days). The patient recovered from the anesthesia smoothly, sating well on room air with no bronchospasm, and patient transferred to her room for an overnight stay. On the first postoperative day, the patient was complaining of chest pain otherwise her vital signs were within normal range, painkiller given to patient with release of garment, symptoms improved temporarily. 4 h later symptoms recur accompanied with tachypnea (respiratory rate: 28) and tachycardia (pulse rate: 120 bpm), the Oxygen saturation was maintained on room air. Upon chest auscultation, diminished air entry was markedly noted on her left side, immediate chest X-ray and electrocardiogram (ECG) was done, which showed unremarkable X-ray and ECG shows sinus tachycardia. Computed tomography which showed left side pneumothorax. An urgent thoracic surgery consultation was done and chest tube was inserted. The patient reported immediate improvement of her symptoms and the vital signs retain to normal range. The patient start ambulating, regular respiratory exercises using the incentive spirometer, and cough exercises to help with chest expansion. On day 3, air leak stopped, chest tube was clipped by thoracic surgery, and the chest tube was removed 24 h later. The patient had a relatively smooth recovery with no other complications.

## Discussion

The most commonly performed cosmetic procedure is liposuction. It is an outpatient procedure that can be performed under local anesthesia in cosmetic centers. Liposuction was firstly known in 1929; with tumescent liposuction as the gold standard [3]. Its use and development made the procedure safe and easy. In spite of that, it’s not a risk free and its complications are underestimated and underreported by patients and caregivers [4]. However, there is still ample literature on the complications associated with liposuction that necessitate the attention toward them to spread the awareness and to engage more regulations. The mortality rate reported is 19–20 per 100,000 which is higher than the mortality rate reported for motor vehicle accidents (16 per 100,000) [5]. Although that pneumothorax is recognized as a rare complication of liposuction, the exact incidence still unknown, and reports have been only limited to small case series and case reports [2–4]. Possible risk factor for the development of pneumothorax in this case is liposuction near the axilla and posterior chest, other risk factors include use of flexible infiltration cannula, and scarring from previous liposuctions. Although this patient has developed pneumothorax, radiograph was unremarkable. This might be due to minimal pleural air or related to patient position. On the basis of the results of this article and review of the literature [2–5], we recommend to include pneumothorax as a potential complication during informed consent, using a stiff > 3.5 mm infiltration cannula, minimizing positive pressure ventilation, emphasized cannula tip awareness in all patients but particularly in patients with previous liposuction or scar tissue, increased caution when operating in the axilla [2], and minimize patient change of position during the procedures.

## Conclusion

Pneumothorax have possibility to happen with liposuction, awareness of possible risk factors should detect by plastic surgeon, to manage earlier as soon as possible.

## Data Availability

Data sharing is not applicable to this article as no datasets were generated or analyzed during the current study.

## Abbreviations

ECG: Electrocardiogram
CT: Computed tomography
PAL: Power-assisted liposuction
UAL: Ultrasound assisted liposuction
VTE: Venous thromboembolism
LMWHs: Low-molecular-weight heparins
